# Urgent Treatment of Complicated Ulcerated Infantile Hemangioma with β-Blockers via Telemedicine: A Case Report

**DOI:** 10.1089/tmj.2023.0338

**Published:** 2024-03-06

**Authors:** Olga Bogomolets, Magdalena Wierzbik-Strońska, Roman Hryshchenko, Catherine Bogomolets

**Affiliations:** ^1^ Faculty of Medicine, Academy of Silesia in Katowice, Zabrze, Poland.; ^2^ Bogomolets Clinic, Kyiv, Ukraine.

**Keywords:** *complicated infantile hemangioma*, *ulceration*, *telemedicine*, *topical timolol*

## Abstract

**Introduction:**

Infantile hemangiomas (IH) exacerbated by ulceration invariably necessitate hospitalization, although simple IHs are sometimes managed remotely. Furthermore, according to international regulations, β-blocker medication for such hemangiomas should be systemic and performed in a clinic, especially if there is infection and risk of bleeding.

**Case:**

War in Ukraine made it impossible to hospitalize and properly examine a patient with a complex ulcerated and infected IH, forcing us to administer β-blocker timolol therapy only through telemedicine.

**Conclusions:**

Our case demonstrates the possibility of successful distant treatment of IH with ulcer using only a topical β-blocker carried out remotely through telemedicine, which is critical in the context of the COVID-19 pandemic, war, hostilities, or natural disasters where inpatient treatment is not available.

## Introduction

Big infantile hemangiomas (IH), complicated by ulceration, usually requires hospitalization.^1^ The therapy of such IH with β-blockers should be systemic and carried out in a clinic.^2–4^ The war and military conditions made evacuating, examining, and hospitalizing a patient impossible, forcing us to perform remote treatment.

Telemedicine became crucial during the war in Ukraine when millions of people appeared in the war zone in the occupied territory and had limited access to hospitals.^5^ Medical assistance with the use of telemedicine gives the possibility of providing the patient with medical services for counseling, diagnosis, and treatment using remote communication tools in the form of electronic information exchange, including through the transmission of the electronic message and conducting video conferences. Due to the war in our case of complicated ulcerated IH, the patient received urgent e-consultation and effective treatment via teledermatology.

According to the PubMed and MEDLINE search, there were no reports of similar treatment (request made using keywords “complicated infantile hemangioma, ulcer, telemedicine, teledermatology”), therefore, we consider our clinical case to be the first experience of such therapy.

In this article, we aimed to describe the successful method of the remote treatment of IH with an ulcer using the topical nonselective β-blocker timolol and to focus on this method of therapy as an option when inpatient treatment is impossible.

## Methods

**Fig. 1.**
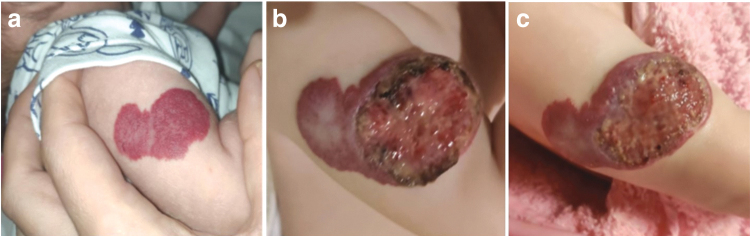
**(a)** First photo of the formation on the child's shoulder, taken by the parents at the age of 2 months and 3 days, non-ulcerated; **(b)** photo at the time of the referral to the clinic. Hemangioma is horizontally located and has the appearance of an elongated, raised lesion of an uneven crimson-raspberry color, with an extensive ulcer with granulation and a partially dried scab, that is torn off in some places with the formation of a bleeding wound; **(c)** positive changes in a wound state after 5 days of the antibiotic treatment and antiseptic dressings, the wound is cleaned of crusts and the wound surface is opened.

The parents consulted the dermatology online service when the baby girl was 3.5 months old. They complained of a bright red tumor with a crusted ulcer with a smell and bleeding. After birth, the child was examined in the maternity hospital and found to be healthy. Hemangioma appeared at 2 months and was treated by parents with alternative medicine—compresses according to folk recipes (castor oil, baths with a bur-marigold, etc.) as a result of which an ulcer formed with crusts that periodically bled (*Fig. 1a*).

At the time of the first e-consultation, the IH spread to a third of the right shoulder (*Fig. 1b*) and was complicated with ulceration and secondary infection. Further examination and hospitalization were recommended. Due to the permanent shelling of the hospital and occupation by enemy troops, hospitalization or evacuation was impossible. Teledermatology was the only way of treatment. Informed Patient consent was received.

## Results

When the parents turned to the clinic, IH complicated with an ulcer in the proliferation phase was diagnosed and secondary infection of the wound was recommended and additional examination (consultation with a cardiologist, ultrasound of the heart, general blood, and urine) and hospitalization to start antibiotic therapy and the use of β-blockers.

But when the parents applied for hospitalization at their place of residence, due to the shelling of the hospital, destruction, and occupation, the child's hospitalization was refused. As far as transportation of the child was impossible due to shelling, online teledermatology treatment was the only method of treatment.

Prescribed: Azithromycinum suspension per oral q.d. 2.5 mL and b.i.d. daily dressing with Grassolind–Octenisept for 5 days (treatment was chosen from what was available in the local pharmacies). The condition of the wound was monitored daily with the help of photos or videos taken by the parents. From the sixth day, topical treatment with β-blockers was started. Three drops of β-blocker timolol 0.5% sterile solution to the IH ulcerated surface plus one to two drops for the unulcerated part b.i.d. Hydrocoll bandage on top. Everyday photo control of treatment. Parents received instructions on the photo and video fixation and were advised to check pulse, blood pressure, and blood sugar levels daily after starting timolol.

**Fig. 2.**
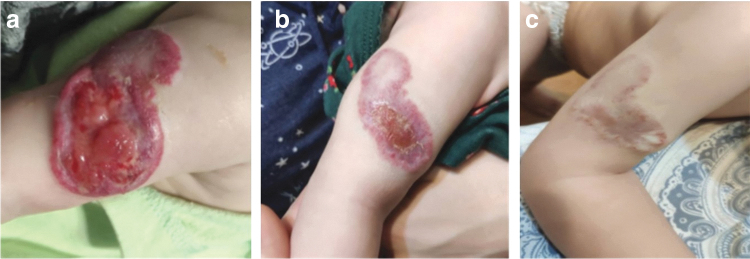
**(a)** Noticed a pause in the dynamics of wound healing in the fourth month of online treatment. Hemangioma demonstrates cleaning and granulation of the wound surface, with epithelialization at the edges, but without further progression; **(b)** photo taken at 8 months of online treatment shows gradual re-epithelialization, a decrease in the size of the hemangioma and a color change; **(c)** photo was taken 6 months after the end of timolol treatment and after one session of laser therapy.

During treatment, a gradual improvement of the wound condition was recorded (*Fig. 1c*). According to parents, during treatment, side effects were absent. In the fourth month of treatment, we marked a lack of positive dynamics and decided to stop using hydrocolloid bandages (*Fig. 2a*). After keeping the wound open, healing continued (*Fig. 2b*).

After 8 months of treatment, complete re-epithelialization was achieved, timolol treatment was continued until the hemangioma completely regressed (*Fig. 2c*).

## Discussion

According to international protocols, the first-line drug in the treatment of ulcerated IH is systemic nonselective β-blockers,^3,6^ but requires examination and hospitalization.^2,4^ In our case, we performed the topical treatment with β-blocker dripped directly into the ulcer, so the level in the blood was probably comparable to that with systemic use; therefore, parents were advised to control blood sugar and pulse. During the entire treatment, these indicators remained within the normal range. Other possible side effects were also not observed. Furthermore, parents noted a lower level of ulcer pain when using topical β-blockers, which is also confirmed in another clinical case.^7^

The mechanism of treatment is based on inhibition of angiogenesis, and induction of apoptosis. It is suggested that it also affects and accelerates the transformation of hemangioma stem cells into adipocytes and affects the renin-angiotensin system.^8–10^ Regarding the possible ulcer recurrence after stopping treatment, as reported by Chang and Kang in cases of using topical propranolol, there were no recurrences at all, unlike other treatment methods.^11^ At the same time, it is necessary to educate parents to reduce the fear of side effects associated with treatment with β-adrenoceptor blockers, as the lack of timely treatment later causes irreversible changes and cosmetic defects, and to continue developing the skills of parents to disseminate remote treatment.

## Conclusions

In our clinical case, effective treatment with antibiotics and topical β-blocker was demonstrated in ulcerated IH via telemedicine. Our forced experience was the result of military action, but we suppose that it shows possibilities for the remote treatment of complicated ulcerated IH, which is extremely important in the conditions of pandemics, war, or natural disasters. Although, it is worth noting that successful teledermatology requires a particularly responsible attitude of parents, their awareness of the peculiarities of telemedicine, and their careful implementation of the doctor's recommendations.
